# Application of Dispersive Liquid-Liquid Microextraction with Graphite Furnace Atomic Absorption Spectrometry for Determination of Trace Amounts of Zinc in Water Samples

**DOI:** 10.1155/2013/743792

**Published:** 2013-05-12

**Authors:** Ali Mazloomifar

**Affiliations:** Department of Chemistry, Shahre Ray Branch, Islamic Azad University, Tehran 18155-144, Iran

## Abstract

A selective and simple method for separation and preconcentration of zinc ions was developed by using dispersive liquid-liquid microextraction. Parameters that have an effect on the microextraction efficiency such as volume of extraction and disperser solvent, extraction time, and adding salt were investigated. Under optimum conditions, a preconcentration factor of 250 was obtained. The limit of detection (LOD) obtained under the optimal conditions was 0.09 ng mL^−1^. The linearity of method was obtained in range of 0.2–50 ng mL^−1^ with a correlation coefficient (*r*) of 0.9974. The relative standard deviation for 10 replicate determinations at 1.0 ng mL^−1^ of zinc was 2.53%. The proposed method was successfully applied to the analysis of zinc in water sample.

## 1. Introduction

Zinc is an essential trace element for humans, animals, plants, and microorganisms. The zinc content in humans is 2–4 g [1]. Zinc plays many fundamental roles in cell replications, gene expression, and in the metabolism of nucleic acid and different proteins [2]. The sensitive, selective, and rapid methods for the determination of zinc are in great demand. Atomic absorption spectrometry (AAS) [3], graphite furnace atomic absorption spectroscopy (GF-AAS) [4], neutron activation analysis (NAA) [5], inductively coupled plasma-atomic emission spectroscopy (ICP-AES) [6], inductively coupled plasma-mass spectrometry (ICP-MS) [7], direct current plasma atomic emission spectrometry (DC-PAES), and X-ray fluorescence are widely applied to the determination of zinc at trace level. Preconcentration technique allows the improvement of detection limit as well as the selectivity of the method.

Sample preparation is one of the most important and crucial steps in the whole analytical process. It is often also the bottleneck for rapidly obtaining the desired results, especially for the determination of trace analytes in samples with complex matrix. For the preconcentration and sample preparation several different microextraction methods have been developed, including liquid-liquid extraction [8, 9], solid phase extraction (SPE) [10], sorption and chelating ion exchange [11], solid phase microextraction [12], homogeneous liquid-liquid extraction [13, 14], and dispersive liquid-liquid microextraction (DLLME) [15–17]. In this work, a dispersive liquid-liquid microextraction coupled with graphite furnace atomic absorption spectrometry (GFAAS) has been developed and optimized for the extraction and determination of zinc. The method is based on chemical complexation of zinc(II) by Phenanthraquinone monophenyl thiosemicarbazone (PPT). DLLME technique was applied for the complex extract, and GFAAS was used to analyze the extracted product.

## 2. Experimental

### 2.1. Reagents and Materials

All the reagents and materials were purchased from Merck (Darmstadt, HE, Germany).

A stock solution of Zn^2+^ ions (1000 *μ*g mL^−1^) was prepared by dissolving an appropriate amount of Zn(NO_3_)_2_·6H_2_O and diluted with doubly distilled water. Working standard solution was obtained daily by stepwise dilution of the standard stock solution.

Phenanthraquinone monophenyl thiosemicarbazone was synthesized using a previously reported method [18]. A standard solution (1.0 mol L^−1^) was prepared by dissolving an appropriate amount of PPT in CCl_4_. Working solutions were prepared by appropriate dilution of the stock solution.

### 2.2. Instruments

A PG Instruments (Leicestershire, UK) model PG990 atomic absorption spectrophotometer was used for the analysis with the appropriate zinc hollow cathode lamp. All measurements were based on integrated absorbance and performed at 307.9 nm for zinc. The furnace program for determination of zinc(II) is shown in Table 1.

### 2.3. Dispersive Liquid-Liquid Microextraction Procedure

For DLLME under optimum conditions, 5 mL analyte solution containing zinc was placed in a 10 mL screw cap glass test tube. Then, 1 mL of ethanol (as disperser solvent) and 200 *μ*L of CCl_4_ (as extraction solvent) containing PPT were rapidly injected into a sample solution by using a microsyringe. A cloudy solution that consisted of very fine droplets of CCl_4_ dispersed into aqueous sample was formed, the solution was gently shaken in a shaker for 10 min, and the analytes were extracted into the fine droplets. After centrifugation at 3000 rpm for 5 min, a small droplet of CCl_4_ phase was sedimented at the bottom of the centrifuge tube. After removal of the whole aqueous solution, 20 *μ*L of the extraction phase was injected into the pyrographite furnace of atomic absorption spectrometry and the zinc concentration was determined by using a hallow cathode lamp of zinc at 307.9 nm.

## 3. Results and Discussion

### 3.1. Optimization of the Experimental Conditions of the Method

Some preliminary experiments were carried out in order to investigate the quantitative extraction of zinc(II) ions using the PPT reagent in the absence of the metal ions. The optimum conditions for extraction of zinc(II) were established by varying the experimental parameters, such as pH of aqueous phase, concentration of ligand, disperser solvent volume, extraction solvent volume, and ionic strength.

#### 3.1.1. Effect of pH

The pH of the aqueous solution is an important factor in DLLME of Zn(II) using PPT because this parameter is directly related to the formation of metal-ligand complex. In this study, the effect of the pH aqueous solutions on the extraction of zinc(II) were examined in the range of 2–10.

**Figure 1:**
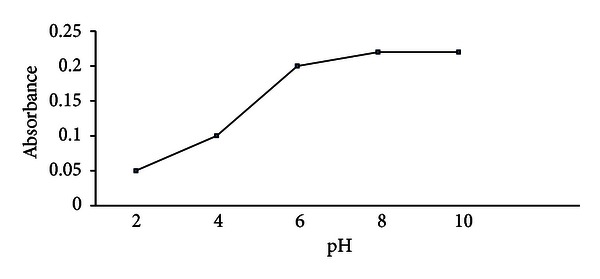
The effect of pH on the absorbance of the system, conditions: sample volume 5.0 mL containing 1.0 ng mL^−1^ of Zn(II), dispersive solvent 1.0 mL ethanol, extraction solvent 100 *μ*L CCl_4_ containing 1.0 × 10^−6^ mol L^−1^of PPT, extraction time 10 min.

According to the results shown in Figure 1, the maximum absorbance was achieved at pH 7.5 and remained nearly constant at higher pH 7.5. Thus, the value of pH 8 was selected for the following experiments.

#### 3.1.2. Effect of Ligand (PPT) Concentration

**Figure 2:**
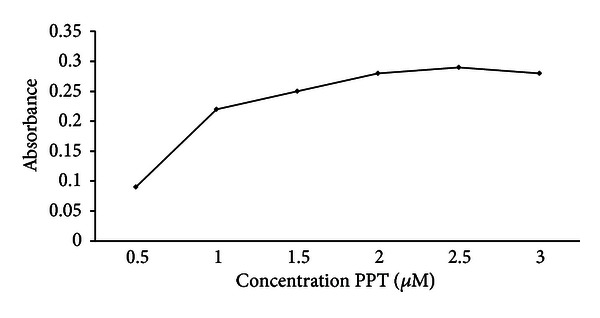
The effect of ligand (PPT) concentration on the absorbance of the system, conditions: sample volume 5.0 mL containing 1.0 ng mL^−1^ of Zn(II), dispersive solvent 1 mL ethanol, extraction solvent 100 *μ*L CCl_4_ containing PPT, extraction time 10 min.

The influence of the ligand concentration was studied by extracting zinc(II) ions with different amounts of the PPT reagent. As shown in Figure 2, the maximum absorbance was achieved at 2.0 × 10^−6^ mol L^−1^ and remained nearly constant at higher concentrations. Thus, a concentration of 2.5 × 10^−6^ mol L^−1^ was applied in the proposed method.

#### 3.1.3. Effect of Extraction Solvent Volume

**Figure 3:**
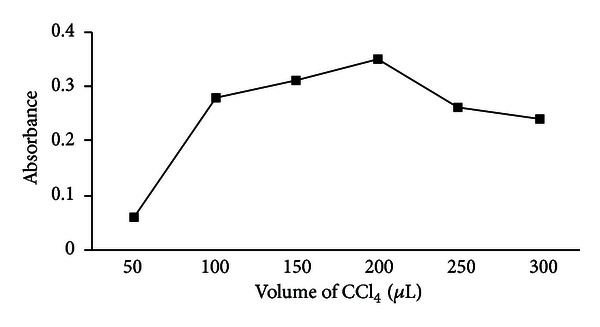
Effect of the extraction solvent volume on the analytical responses, conditions: sample volume 5.0 mL containing 1.0 ng mL^−1^ Zn(II), dispersive solvent 1 mL ethanol, extraction solvent CCl_4_ containing 2.5 × 10^−6^ mol L^−1^ PPT, extraction time 10 min.

The extraction solvent volume has great effects on enrichment factor. In order to examine the effect of the extraction solvent volume, solutions containing different volume of CCl_4_ (50, 100, 150, 200, 250, 300 *μ*L) were subjected to the same DLLME procedure. When the volume of extraction solvent was increased, the volume available for the measurement also increased, but the enrichment factor decreased.  Figure 3 shows the variation of absorbance versus volume of the extraction solvent. Therefore, 200 *μ*L of extraction solvent was selected as volume optimum.

#### 3.1.4. Effect of the Disperser Solvent Volume

**Figure 4:**
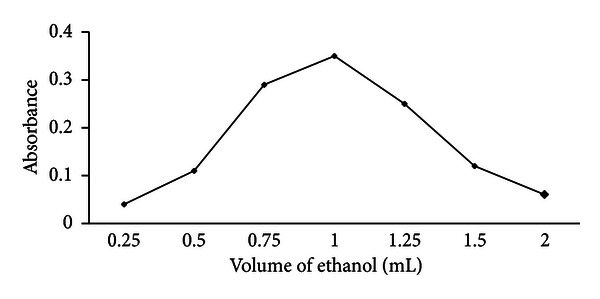
Effect of the disperser solvent volume on analytical responses, Conditions: sample volume 5.0 mL containing 1.0 ng mL^−1^ Zn(II), extraction solvent 200 *μ*L CCl_4_ containing 2.5 × 10^−6^ mol L^−1^ PPT, extraction time 10 min.

The effect of the volume of the disperser solvent was investigated by changing ethanol volume to 0.25, 0.50, 0.75, 1.0, 1.25, 1.5, and 2.0 mL, respectively. The results are shown in Figure 4. According to Figure 4, absorbance increases first and then decreases by increasing the volume of ethanol. The reason could be that at a low volume of ethanol, a cloudy state could not be well formed, therefore resulting in a low absorbance. At a higher volume of ethanol, absorbance decreases because of decreasing distribution coefficient. A 1.0 mL volume was selected as an optimum volume for disperser solvent.

#### 3.1.5. Effect of Extraction Time

Extraction time is one of the most important factors in DLLME as most extraction procedures. The extraction time is defined as the time interval between the addition of the mixture of disperser solvent (ethanol) and extraction solvent (CCl_4_) containing PPT to the sample and the start of centrifugation.

**Figure 5:**
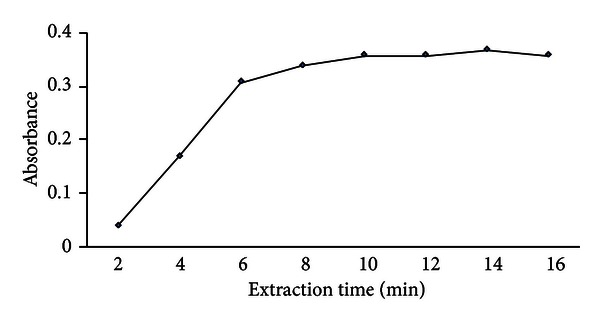
Effect of the extraction time on the analytical responses, conditions: sample volume 5.0 mL containing 1.0 ng mL^−1^ Zn(II), dispersive solvent 1.0 mL ethanol, extraction solvent 200 *μ*L CCl_4_ containing 2.5 × 10^−6^ mol L^−1^.

After the addition of the mixture, the solution was gently shaken in a shaker for an appropriate time before centrifugation. Effect of extraction time was studied in the range between 2 and 16 min. The results are shown in Figure 5. According to the obtained results, the absorbance reaches its maximum value at 10 min and then remains approximately constant with further increasing of time. A 10-minute time was selected as an optimum time.

#### 3.1.6. Effect of Ionic Strength

Effect of salt addition on extraction efficiency of DLLME was studied with the NaCl concentration in the range 0.0–5.0 (w/v%). No significant impact on the analytical signal was observed. Hence, NaCl was not added in all subsequent extraction experiments.

### 3.2. Analytical Performance

Under the optimized experimental conditions, linear range, calibration curve, detection limit, preconcentration factor, and precision were obtained (Table 2). The calibration curve was linear in the range of 0.20–50 ng mL^−1^ of Zn(II).

The preconcentration factor was defined as the ratio between the analyte concentration in the sedimented phase (C_sed_) and the initial concentration of the analyte (C_0_) in the aqueous sample.

The relative standard deviation (RSD) for 10 replicate measurements of 1.0 ng mL^−1^ zinc(II) was 2.53%.

The limit of detection was defined as the concentration of analyte giving signals equivalent to 3 times the standard deviation of the blank plus the net blank signal.

The extraction recovery was defined as the percentage of total analyte which was extracted in the sedimented phase.

### 3.3. Selectivity

The effects of common coexisting ions on the recovery of zinc(II) were studied. In these experiments, 5 mL of solution contains 1.0 ng mL^−1^ of zinc and various amounts of interfering ions were treated under the optimum conditions. The tolerance limit was defined as the concentration of the added species caused less than ±5% relative error. The results obtained are shown in Table 3. 

### 3.4. Application

The proposed DLLME-GFAAS was applied for the determination of zinc in several water samples. These results, as the average of five separate determinations, are shown in Table 4. The proposed method gave satisfactory average recoveries.

## 4. Conclusions

Sample preparation by dispersive liquid-liquid microextraction is a method that is considered inside the green chemistry, because of the small volumes of dissolvent employed. This study proposes the use of DLLME as a method for extraction and preconcentration of zinc as a prior step to its determination by GFAAS.

## Figures and Tables

**Table 1:** Heating program for determination of zinc.

Step	Temperature (°C)	Ramp (s)	Hold (s)	Ar flow rate (mL min^−1^)
1	80	5	20	250
2	200	5	20	250
3	800	5	20	250
4	1800	0	4	0
5	2100	1	2	250

**Table 2:** Regression and analytical parameters.

Regression equation using DLLME	*A* = 0.016 + 0.344 *C*
Linear range	0.20–50 ng mL^−1^
Limit of detection	0.09 ng mL^−1^
Preconcentration factor	250
*r*	0.9974
RSD% (*n* = 10)	2.53
Extraction recovery%	98.6

**Table 3:** Influence of foreign ions.

Ions	Tolerance ratio
Ca^2+^, Ba^2+^, Mg^2+^, Mn^2+^, K^+^, Na^+^, Ni^2+^, SO_4_^2−^, NO_3_^−^, Cl^−^, NO_2_^−^	500 : 1
Fe^2+^, PO_4_^3−^, Hg^2+^, Cu^2+^, HCO_3_^−^, Ag^+^, Pb^2+^	300 : 1

**Table 4:** Determination of zinc(II) in water samples.

Sample	Spiked (ng mL^−1^)	Measured (ng mL^−1^)	RSD% (*n* = 5)	Recovery%
Well water	0	nd*	—	—
	5	4.82	2.86	96.4

Tap water	0	nd*	—	—
	5	4.78	2.25	95.6

*nd: not detected.
